# Venous Thromboembolism Risk Score and Pregnancy

**DOI:** 10.3389/fcvm.2022.863612

**Published:** 2022-04-11

**Authors:** Tiphaine Raia-Barjat, Osasere Edebiri, Céline Chauleur

**Affiliations:** ^1^Department of Gynecology and Obstetrics, Centre Hospitalier Universitaire de Saint-Étienne, Saint-Étienne, France; ^2^INSERM U1059 SAINBIOSE, Université Jean Monnet, Saint-Étienne, France; ^3^Departments of Haematology, Mater Misericordiae University Hospital and Rotunda Hospital, Dublin, Ireland

**Keywords:** venous thromboembolism, pregnancy, risk score, guidelines, thrombosis

## Abstract

Venous thromboembolism (VTE) is a major contributor to maternal morbidity and mortality worldwide. Pregnancy is associated with the development of a baseline hypercoagulable state. The two strongest risk factors for pregnancy-associated VTE are previous VTE and/or high risk thrombophilia. The others risk factors for VTE during pregnancy are well known such as maternal, pregnancy and delivery characteristics. Considering the variation in recommendation in guidelines and low-quality evidence on the prevention, diagnosis and treatment, practice differs between countries and clinical institutions. Some authors developed risk scores, enabling individualized estimation of thrombotic risk during pregnancy, and permitting implementation of a risk-adapted strategy for thromboprophylaxis during pregnancy and postpartum. This review describes the existing VTE risk scores during the antenatal and postnatal period. The important message beyond the score used is that all women should undergo VTE risk factor assessment. The use of a Computerized Clinical Decision Support System for VTE risk assessment should be explored in obstetrics.

## Introduction

Pregnancy is a physiological hypercoagulable state (1, 2), such that venous thromboembolism (VTE) is a major contributor to maternal morbidity and mortality accounting for 13.8% of maternal deaths in developed regions and 3.2% worldwide (3). The absolute incidence of VTE in pregnancy is 1 or 2 cases per 1,000 pregnancies, with 1 death per 100 000 pregnancies. Pulmonary embolism (PE) is one of the three leading causes of maternal death (4, 5). The risk of VTE was 5-fold increased during pregnancy and 60-fold increased during the first 3 months after delivery compared with non-pregnant women (4, 6). The risk factors for VTE during pregnancy are well-known such as maternal characteristics (age, BMI, thrombophilia, tobacco, co-morbidities…), pregnancy characteristics (twin pregnancy, preeclampsia…) and delivery characteristics (cesarean section, hemorrhage…) (7, 8). Pre-existing and acquired factors throughout pregnancy mean that the risk is individual and evolving over time. The cumulative weight of these risk factors made it possible to establish prediction scores for the occurrence of VTE during the antenatal or postnatal period. These scores make it possible to initiate thromboembolic prophylaxis and prevent the occurrence of VTE. However, there are many scores and recommendations on the subject which can be confusing for clinicians. Care was considered non-optimal in 59% of deaths caused by VTE complication and the rate of preventability was 34.8% (9). The important message beyond the score used is that all women should undergo VTE risk factor assessment continuously before, during and after pregnancy (10, 11).

### Thromboembolic Risk Change by Pregnancy State

The mechanisms of venous thrombosis were described by Virchow, and describe three etiopathogenic components: venous stasis, hypercoagulability and tissue damage. These three mechanisms are often concomitant, the role of each being more or less prevalent (12). Pregnant women have all components of Virchow's triad (13). Venous stasis is secondary to physiological vasodilatation and compression of the vena cava and left common iliac by the gravid uterus (14). Pregnancy is a physiological hypercoagulable state secondary to the increase of clotting factor concentrations, inhibition of fibrinolysis and a reduction in anticoagulant agent levels (15). Finally, tissue damage occurs with endothelial damage to the pelvic vessels during delivery.

Thrombotic events occur throughout pregnancy, with half occurring in the antenatal period and half in the postnatal period (8). VTEs correspond to deep vein thrombosis 3 times higher than pulmonary embolism in pregnancy (4). Two-thirds of deep vein thrombosis occur in the antenatal period while two-thirds of pulmonary embolism occur in the postnatal period (16). Approximately 80% of postpartum thromboembolic events occur in the first 3 weeks after delivery (8, 17). An increased risk persists until 12 weeks after delivery (18).

Deep-vein thrombosis in pregnant women occurs more frequently in the left leg (85%) compared to those in non-pregnant individuals (55%), and is more often proximal with 72% in the iliofemoral veins compared to 9% in those who are not pregnant (19). Pregnant women have also a greater risk of embolic complications and post-thrombotic syndrome (20).

### Risk Factors for Venous Thromboembolism During Pregnancy

The two strongest risk factors both in antenatal and postnatal period are previous VTE and thrombophilia (11). An odds ratio (OR) of 24.8 (95% confidence interval [CI] 17.1–36) for previous VTE, 51.8 (95% CI 38.7–69.2) for thrombophilia and 15.8 (95% CI 10.9–22.8) for antiphospholipid syndrome were reported in a large study (5). The risk of VTE recurrence during pregnancy is increased 3.5 times compared with recurrence in the non-pregnant period. This risk of recurrence appears to be constant over the whole course of pregnancy (21). In pregnancy, the risk of recurrence is very low if VTE was provoked by transient risk factors such as surgery, trauma, or immobility unrelated to estrogen or pregnancy. The risk of recurrence is greater if previous VTE was unprovoked due to no identified precipitating factor present, or if prior VTE was in pregnancy or associated with hormonal contraception (22, 23). Increased VTE risk depending on the type of thrombophilia, the association with personal or family VTE history and additional risk factors. The Royal College of Obstetricians and Gynecologists (RCOG) defined low risk thrombophilia as heterozygous for factor V Leiden or prothrombin G20210A mutations; and high risk thrombophilia as antithrombin deficiency, protein C or S deficiency, compound or homozygous for low-risk thrombophilia (24). A recent meta-analysis confirmed that the absolute risk of VTE exceeded 3% only for women with antithrombin, protein C, and protein S deficiencies, or homozygosity for factor V Leiden (25). The risk of VTE in women with antiphospholipid syndrome without VTE history is poorly described but seems increased and to be considered as a low risk thrombophilia (5, 24).

The other risk factors could be divided into maternal, pregnancy and delivery characteristics. Maternal characteristics that moderately influence the VTE risk are body mass index (BMI) ≥40 kg/m^2^, or 25 kg/m^2^ with antepartum immobilization and a medical co-morbidity like sickle cell disease or preexisting diabetes (26, 27). Maternal characteristics associated with low increase VTE risk are: age ≥35 years, BMI ≥25 to 40 kg/m2, parity ≥3, smoking, assisted reproductive technology, varicose veins and family history of VTE. Pregnancy characteristics such as, hospital admission, surgery, immobility/long-distance travel, systemic infection, and ovarian hyper stimulation syndrome moderately influence VTE risk. Hyperemesis, multiple gestation, intrauterine growth restriction, and preeclampsia have a small influence on risk. For delivery characteristics, emergency cesarean section and postpartum hemorrhage >1,000 mL or blood transfusion moderately increase risk. Stillbirth, preterm delivery <37weeks' gestation, prolonged labor >12 h, planned cesarean delivery, operative vaginal delivery and manual removal of the placenta are mild risk factors (28). The weight of each of these risk factors differs between studies. More than three-quarters of women had at least 1 VTE risk factor (78%) and more than 40% had multiple (2 or more) VTE risk factors (29).

### Venous Thromboembolism Risk Score During Pregnancy

Some authors developed risk-scoring systems, enabling individualized estimation of thrombotic risk during pregnancy, and permitting implementation of a risk-adapted strategy for anti-thrombotic prophylaxis during pregnancy and puerperium. We have found seven scores allowing an assessment of the VTE risk during pregnancy and the postpartum period (Table 1). In these studies, individual risk factors were allocated a weighted score. Variations among these scores exists in their development, target population, risk factors and the weight of risk assigned to each risk factor.

**Table 1:** Venous thromboembolism risk score during pregnancy and postpartum period.

**Antenatal and postnatal risk scores**							
**Scores**	**Lindqvist et al**. **(****30****)**	**Dargaux et al**. **(****31****–****33****)**	**Chauleur et al**. **(****34****–****36****)**	**Schoenbeck et al**. **(****37****)**	**RCOG** **(****24****)**	**Testa et al**. **(****38****)**	**Chau et al**. **(****39****)**
**First published**	**2002–2011**	**2005**	**2008**	**2011**	**2015**	**2015**	**2019**
**Country**	**Sweden**	**France**	**France**	**United Kingdom**	**United Kingdom**	**Italy**	**France**
**Population**	**High risk VTE**	**Thrombophilia and/or a VTE history**	**High risk of VTE**	**High risk of VTE**	**Unselected**	**Unselected**	**Unselected**
**Personal history of VTE**							
Recurrent personal VTE events	Very high risk	3	12				6
VTE during childhood		6					6
VTE in previous pregnancy, cerebral VTE, or massive pulmonary embolism	≥4	6	6	2	4	3	6
Spontaneous or estrogen-induced or proximal VTE	≥4	3	6	2	4	3	6
Spontaneous or estrogen-induced distal DVT	≥4	2	3	2	4	3	6
Proximal VTE with transitory risk factor	≥4	2	3	1	3	3	3
Distal VTE with transitory risk factor	≥4	1	0	1	3	3	3
Residual venous thrombi with clinical signs of post-thrombotic syndrome		3	0				
Recent VTE history <2 years		2					
**Familial history of VTE**							
Family history (1st degree) of proximal VTE without risk factors	1		2		1	1	3
Family history (1st degree) of proximal VTE recurrent or severe	1	1	2	0.5 (two or more)		1	
Family history of non-severe VTE: distal or triggering factor or >60 years			0			1	
**Thrombophilia**							
Antithrombin III deficiency	Very high risk	1	10	3	3	3	3
**Scores**	**Lindqvist et al**. **(****30****)**	**Dargaux et al**. **(****31****–****33****)**	**Chauleur et al**. **(****34****–****36****)**	**Schoenbeck et al**. **(****37****)**	**RCOG** **(****24****)**	**Testa et al**. **(****38****)**	**Chau et al**. **(****39****)**
**First published**	**2002–2011**	**2005**	**2008**	**2011**	**2015**	**2015**	**2019**
**Country**	**Sweden**	**France**	**France**	**United Kingdom**	**United Kingdom**	**Italy**	**France**
**Population**	**High risk VTE**	**Thrombophilia and/or a VTE history**	**High risk of VTE**	**High risk of VTE**	**Unselected**	**Unselected**	**Unselected**
Protein C or protein S deficiency	2	3	4	C: 1.5, S: 1	3	3	1
Factor V Leiden or prothrombin G20210A (factor II) homozygosity	3	1	5	3	3	3	3
Factor V Leiden or prothrombin G20210A (factor II) heterozygosity	1	3	3	1	1	2	1
Combined thrombophilia		1	4		3	3	3
Obstetrical Antiphospholipid syndrome	≥4		9	1	1	3	6
**Maternal, pregnancy, and delivery characteristics**							
Age (>35 years), Obesity, Parity ≥3, Smoking, varicose veins, Multiple pregnancy	Age>40: 1 Obesity: 1	Age >35 years: 1 Obesity:1 Multiple pregnancy:1	Parity >4: 0 Varicose veins: 0	Age>35: 0.5 Obesity: 0.5	1 for each Except BMI≥30: 1 ≥40: 2	Age>35: 0.5 Obesity: 1 Varicose veins: 0.5 Parity >4: 0.5 HTA/diabète:0.5	1 for each
Heterozygous sickle-cell trait, Inflammatory bowel disease, Nephrotic syndrome, Lupus	IBD: 1		Lupus: 0		3	2 for each	1 for each
OHSS (first trimester only)					4	1	
Hyperemesis					3	0.5	
IUGR, PE, placental abruption, ART	PE: 1 Placental abruption: 1				PE: 1 ART: 1	PE: 0.5	IUGR:1 PE: 1
Bed rest, immobilization, Sepsis	Bed rest: 2	Bed rest: 2			1 for each	Bed rest: 3 sepsis: 1	2 for each
Emergency cesarean delivery	1				2	0.5	2
**Scores**	**Lindqvist et al**. **(****30****)**	**Dargaux et al**. **(****31****–****33****)**	**Chauleur et al**. **(****34****–****36****)**	**Schoenbeck et al**. **(****37****)**	**RCOG** **(****24****)**	**Testa et al**. **(****38****)**	**Chau et al**. **(****39****)**
**First published**	**2002–2011**	**2005**	**2008**	**2011**	**2015**	**2015**	**2019**
**Country**	**Sweden**	**France**	**France**	**United Kingdom**	**United Kingdom**	**Italy**	**France**
**Population**	**High risk VTE**	**Thrombophilia and/or a VTE history**	**High risk of VTE**	**High risk of VTE**	**Unselected**	**Unselected**	**Unselected**
Elective cesarean section, Mid-cavity or rotational operative delivery, Prolonged labor (>24 h), PPH (>1 liter or transfusion), Preterm birth <37 + 0 weeks in current pregnancy, Stillbirth in current pregnancy					1 for each	Blood transfusion: 2	PPH >500 mL: 1
Hemostatic hysterectomy, embolization, or arterial ligation					3		3
Total score	Weighted risk score	Weighted risk score	Weighted risk score	Weighted risk score	Weighted risk score	Weighted risk score	Weighted risk score
Thromboprophylaxis A. 1 conservative management, 2 Compression B. prophylactic LMWH from delivery 1 until 6 weeks postpartum 2 short duration C. prophylactic LMWH from 28 weeks until 6 weeks postpartum D. prophylactic LMWH from diagnosis of pregnancy until 6 weeks postpartum E. Adjusted dose LMWH	0–1: A 2: B2 3: B1 ≥4: D Very high risk: E	<3: B1 3–5: C ≥ 6: D	1–3: B1 4: C 5-11: D ≥12: E	<1.0: A 1.0–1.5: B1 2.0–2.5: C 3.0 or more: D	*Antenatally* 3: C ≥4: D *Postnatally* Low-risk thrombophilia + familial VTE history: B ≥ 2: B2	0–1: A1 1.5–2: A2 ≥2.5: LMWH as described by RCOG	0: A1 1–2: A2 3–5: B1 ≥6: D

*VTE, Venous thromboembolism; IBD, Inflammatory bowel disease, IUGR, intrauterine growth restriction; PE, preeclampsia; ART, assisted reproductive technology; OHSS, ovarian hyper stimulation syndrome; PPH, postpartum hemorrhage; LMWH, low molecular weight heparin; RCOG, Royal college of Obstetricians and Gynecologists*.

Four scores are addressed to a population at high risk of VTE. Lindqvist et al. was the first to propose a risk score for VTE during pregnancy (40, 41). Estimates of absolute risk of pregnancy-related VTE were calculated by multiplying reported prevalence-adjusted odds ratios by the given variables. With this VTE risk estimation, more women at high risk can be identified in the postpartum period. The authors did not detail the decision threshold for thromboprophylaxis, and this unvalidated score cannot therefore be used routinely. The score was modified in Swedish guidelines but was not validated (30). Dargaud et al. proposed a practical risk score called “the Lyon VTE score” (31–33). This score was established according to data from the literature and validated in two prospective studies on 286 and 566 patients with thrombophilia or VTE history. The effectiveness of this score has not yet been demonstrated in clinical practice to reduce the incidence of VTE. Using a Delphi approach, Chauleur et al. developed an easy-to-use tool, the “STRATHEGE score,” enabling individualized estimation of thrombotic risk during pregnancy and permitting implementation of a risk-adapted strategy for anti-thrombotic prophylaxis during pregnancy and postpartum (34–36). The score is intended for pregnant women at risk of VTE and placental vascular complications. In a prospective multicenter before-after study on 2,000 patients at risk, the use of the score reduced the risk of VTE (RR 0.68 [0.55; 0.83]) especially during pregnancy (RR 0.30 [0.14; 0.67]) without any significant increase in bleeding. In the united Kingdom in 2011, a multidisciplinary group of physicians, hematologists, and obstetricians established the “Thromboprophylaxis Scoring System” (37). The scoring system improved the consistency of advice and increased the mean duration of thromboprophylaxis, but no effect on reducing the incidence of VTE could be highlighted because the patient cohort was too small.

Three other scores are addressed to an unselected pregnant population. The RCOG propose a risk assessment for VTE based on adjusted odds ratios for risk factors (24, 42). This score has not been validated, but there is evidence that the implementation of these practice guidelines in the United Kingdom decreased mortality from VTE. Maternal mortality rates decreased from 1.94 deaths per 100,000 births from 2003 to 2005, to 1.01 from 2011 to 2013 (42, 43). A working group of hematologists, internists and gynecologists in Italy created a model to evaluate the risk of VTE in pregnancy called “Pregnancy Health-care Program” (38). The score determined whether or not to initiate heparin thromboprophylaxis, however in the event of required heparin treatment, the score refers to the RCOG recommendations. The score was validated on 1,800 patients in Italy but its effectiveness has not been demonstrated. The most recent score from 2019 is that of Chau et al. The score was validated in a study on 1,000 patients, comparing its effectiveness via, one retrospective period for the population before implementation of the score, and one prospective period post implementation of the score. Use of the VTE risk score at the first consultation in pregnancy increased the likelihood of appropriate treatment (OR 1.5, 95% CI 1.2–1.9; *P* = 0.002) and reduced the risk of undertreatment (OR 0.5, 95% CI 0.4–0.7; *P* < 0.001). No effect on reducing the incidence of VTE could be highlighted.

The feasibility of routine use of the VTE risk score by clinicians and safety in the absence of increased risk of bleeding has been proven. All the VTE risk scores are finally very close. Two studies have shown a promotion of appropriate thromboprophylaxis with the use of VTE risk scores (33, 39). Only one study showed an effectiveness of these risk scores in reducing the incidence of VTE (36). Indirect evidence of the effectiveness of the RCOG score exists through the reduction in VTE mortality seen in the UK.

### Venous Thromboembolism Risk Score in Postpartum Period

All the previous scores allowed the calculation of a score from which followed a course of action to be taken in the postpartum period. Three scores allowing an assessment of the VTE risk only in the postpartum period will be discussed. These scores are for very different populations, with different risk factors considered and with different contribution of each risk factor to the overall risk of VTE. Emergency cesarean delivery, stillbirth, varicose veins, PE/eclampsia, postpartum infection, and comorbidities were the strongest predictors of VTE in the final multivariable model based on data from 433 353 deliveries. The sensitivity of the model to predict VTE is 68% while that of RCOG is only 63% at similar thresholds (44). The disadvantage of this score is that it quantifies absolute risk of postpartum venous thromboembolism and does not give guidance in terms of thromboprophylaxis. The French National College of Gynecologists and Obstetricians proposed a score adapted from existing recommendations. For every cesarean delivery, mechanical thromboprophylaxis with elastic stockings is recommended with or without the addition of LMWH according to the presence of additional risk factors. The score is determined by multiplying the adjusted Odds-Ratio for major and minor risk factors. The treatment is necessary when the combined OR of added risk factors is > 10 (45). The disadvantage of this score is its complex calculation by multiplication. In the recommendations of the American College of Chest Physicians (ACCP) (11), thromboprophylaxis was implemented in the postpartum period when the risk of VTE was >3% (11). None of these scores showed an effectiveness of these risk scores in reducing the incidence of VTE.

## Discussion

Several international organizations have published recommendations on the prevention of VTE during pregnancy by giving priority to prophylaxis in the event of previous VTE and thrombophilia (11, 24, 46, 47). Variation exists in the risk factors considered, the contribution of each risk factor to the overall risk, and the threshold at which a woman is at risk of VTE. It remains unknown whether risk factors are additive or multiplicative. A 5-fold difference in the number of women who would theoretically receive a recommendation for postpartum thromboprophylaxis by various international guidelines was observed, which ranged from 7% under ACOG to 37% under RCOG guidelines (29). These variations could be explained by the low quality evidence on the effectiveness of thromboprophylaxis that led to use expert opinion and consensus-derived guidelines. These discrepancies in the recommendations and their complexity may discourage their routine use by primary care practitioners and gynecologists less familiar with VTE. The utilization of thromboembolism prophylaxis adapted to individualized risk assessment remains unused in many countries (48, 49).

The important message is that it is recommended that all women undergo a documented assessment of risk factors for VTE in early pregnancy or pre-pregnancy. Risk assessment should be repeated if the woman is admitted to hospital for any reason or develops other intercurrent problems (24). Risk assessment should be repeated intrapartum or immediately in postpartum. The National Partnership for Maternal Safety under the guidance of the Council on Patient Safety in Women's Health Care propose a safety bundle organized into four domains, readiness, recognition and prevention, response and reporting and systems learning (1). *Readiness* discusses the use of a standardized VTE risk score (2). *Recognition* is divided into the identification of appropriate patients for thromboprophylaxis, as well as education for patients and health care workers (3). *Response* suggests the use of standardized recommendations for mechanical thromboprophylaxis, dosing of anticoagulation and for appropriate timing of pharmacologic prophylaxis with neuraxial anesthesia. Finally (4) *Reporting and systems learning* recommend to review all thromboembolism events (50).

The introduction of VTE risk scores and electronic health records aims to reduce variation in care and improve the reliability of action in the prevention of VTE. Yet no standardized VTE risk score exists. It seems necessary to differentiate in the scores, high-risk patients (with previous VTE and/or with thrombophilia) from other patients, and to carry out antenatal and postnatal assessments given evolving risk factors. Clinicians do not uniformly use existing risk-stratification tools and, when used, clinicians often use the tools incorrectly, producing an underestimation of a patient's risk for VTE. However, the complexity of the risk assessment signifies the need for an automatic computerized system (51). Some studies have used Computerized Clinical Decision Support Systems (CCDSS) to stratify the patient according to VTE risk and make suggestions for thromboprophylaxis outside the context of pregnancy. A CCDSS is a rule- or algorithm-based software that can be integrated into an electronic health record and uses data to present evidence-based knowledge at the individual patient level. In a systematic review, the use of CCDSS was associated with a 2-fold increase in the rate of ordering prophylaxis for VTE when compared with controls (odds ratio, 2.35; 95% CI, 1.78–3.10; *P* < 0.001) and a significant decrease in the risk of VTE events (risk ratio, 0.78; 95% CI, 0.72–0.85; *P* < 0.001) (51). Further research and data using large study cohorts reporting the use of CCDSS in obstetric settings is required.

## Author Contributions

TR-B wrote the different parts of the review. CC designed the manuscript and supervised the progress of the review. OE verified the English. All authors contributed to the article and approved the submitted version.

## Conflict of Interest

The authors declare that the research was conducted in the absence of any commercial or financial relationships that could be construed as a potential conflict of interest. The handling editor BT declared a shared affiliation, though no other collaboration, with two of the authors TR-B and CC at the time of the review.

## Publisher's Note

All claims expressed in this article are solely those of the authors and do not necessarily represent those of their affiliated organizations, or those of the publisher, the editors and the reviewers. Any product that may be evaluated in this article, or claim that may be made by its manufacturer, is not guaranteed or endorsed by the publisher.

## References

[1] Greer IA Aharon A Brenner B Gris JC. Coagulation and placenta-mediated complications. Rambam Maimonides Med J. (2014) 5:e0034. 10.5041/RMMJ.10168 25386350 PMC4222423

[2] Gris JC Bouvier S Cochery-Nouvellon É Mercier É Mousty È Pérez-Martin A. The role of haemostasis in placenta-mediated complications. Thromb Res. (2019) 181(Suppl 1):S10–4. 10.1016/S0049-3848(19)30359-7 31477220

[3] Say L Chou D Gemmill A Tunçalp Ö Moller A-B Daniels J. Global causes of maternal death: a WHO systematic analysis. Lancet Glob Health. (2014) 2:e323–33. 10.1016/S2214-109X(14)70227-X 25103301

[4] Heit JA Kobbervig CE James AH Petterson TM Bailey KR Melton LJ. Trends in the incidence of venous thromboembolism during pregnancy or postpartum: a 30-year population-based study. Ann Intern Med. (2005) 143:697–706. 10.7326/0003-4819-143-10-200511150-00006 16287790

[5] James AH Jamison MG Brancazio LR Myers ER. Venous thromboembolism during pregnancy and the postpartum period: incidence, risk factors, and mortality. Am J Obstet Gynecol. (2006) 194:1311–5. 10.1016/j.ajog.2005.11.008 16647915

[6] Pomp ER Lenselink AM Rosendaal FR Doggen CJM. Pregnancy, the postpartum period and prothrombotic defects: risk of venous thrombosis in the MEGA study. J Thromb Haemost. (2008) 6:632–7. 10.1111/j.1538-7836.2008.02921.x 18248600

[7] Ewins K Ní Ainle F. VTE risk assessment in pregnancy. Res Pract Thromb Haemost. (2020) 4:183–92. 10.1002/rth2.12290 32110748 PMC7040539

[8] Jacobsen AF Skjeldestad FE Sandset PM. Incidence and risk patterns of venous thromboembolism in pregnancy and puerperium–a register-based case-control study. Am J Obstet Gynecol. (2008) 198:233. 10.1016/j.ajog.2007.08.041 17997389

[9] Rossignol M Rigouzzo A Verspyck É Le Guern V pour le Comité National d'Experts sur la Mortalité Maternelle. [Maternal mortality due to venous thromboembolism in France 2013–2015]. Gynecol Obstet Fertil Senol. (2021) 49:67–72. 10.1016/j.gofs.2020.11.012 33197653

[10] Okoroh EM Azonobi IC Grosse SD Grant AM Atrash HK James AH. Prevention of venous thromboembolism in pregnancy: a review of guidelines, 2000–2011. J Womens Health (Larchmt). (2012) 21:611–5. 10.1089/jwh.2012.3600 22553908 PMC4467199

[11] Bates SM Greer IA Middeldorp S Veenstra DL Prabulos A-M Vandvik PO. VTE, thrombophilia, antithrombotic therapy, and pregnancy: antithrombotic therapy and prevention of thrombosis, 9th ed: american college of chest physicians evidence-based clinical practice guidelines. Chest. (2012) 141:e691S−736S. 10.1378/chest.11-2300 22315276 PMC3278054

[12] Bourjeily G Paidas M Khalil H Rosene-Montella K Rodger M. Pulmonary embolism in pregnancy. Lancet. (2010) 375:500–12. 10.1016/S0140-6736(09)60996-X 19889451

[13] Ramlakhan KP Johnson MR Roos-Hesselink JW. Pregnancy and cardiovascular disease. Nat Rev Cardiol. (2020) 17:718–31. 10.1038/s41569-020-0390-z 32518358

[14] Macklon NS Greer IA Bowman AW. An ultrasound study of gestational and postural changes in the deep venous system of the leg in pregnancy. Br J Obstet Gynaecol. (1997) 104:191–7. 10.1111/j.1471-0528.1997.tb11043.x 9070137

[15] Rodger M Sheppard D Gándara E Tinmouth A. Haematological problems in obstetrics. Best Pract Res Clin Obstet Gynaecol. (2015) 29:671–84. 10.1016/j.bpobgyn.2015.02.004 25819750

[16] Ray JG Chan WS. Deep vein thrombosis during pregnancy and the puerperium: a meta-analysis of the period of risk and the leg of presentation. Obstet Gynecol Surv. (1999) 54:265–71. 10.1097/00006254-199904000-00023 10198931

[17] Tepper NK Boulet SL Whiteman MK Monsour M Marchbanks PA Hooper WC. Postpartum venous thromboembolism: incidence and risk factors. Obstet Gynecol. (2014) 123:987–96. 10.1097/AOG.0000000000000230 24785851

[18] Kamel H Navi BB Sriram N Hovsepian DA Devereux RB Elkind MSV. Risk of a thrombotic event after the 6-week postpartum period. N Engl J Med. (2014) 370:1307–15. 10.1056/NEJMoa1311485 24524551 PMC4035479

[19] Greer IA. Clinical Practice. Pregnancy complicated by venous thrombosis. N Engl J Med. (2015) 373:540–7. 10.1056/NEJMcp1407434 26244307

[20] Wik HS Jacobsen AF Sandvik L Sandset PM. Prevalence and predictors for post-thrombotic syndrome 3 to 16 years after pregnancy-related venous thrombosis: a population-based, cross-sectional, case-control study. J Thromb Haemost. (2012) 10:840–7. 10.1111/j.1538-7836.2012.04690.x 22452811

[21] Pabinger I Grafenhofer H Kyrle PA Quehenberger P Mannhalter C Lechner K. Temporary increase in the risk for recurrence during pregnancy in women with a history of venous thromboembolism. Blood. (2002) 100:1060–2. 10.1182/blood-2002-01-0149 12130523

[22] Iorio A Kearon C Filippucci E Marcucci M Macura A Pengo V. Risk of recurrence after a first episode of symptomatic venous thromboembolism provoked by a transient risk factor: a systematic review. Arch Intern Med. (2010) 170:1710–6. 10.1001/archinternmed.2010.367 20975016

[23] De Stefano V Martinelli I Rossi E Battaglioli T Za T Mannuccio Mannucci P. The risk of recurrent venous thromboembolism in pregnancy and puerperium without antithrombotic prophylaxis. Br J Haematol. (2006) 135:386–91. 10.1111/j.1365-2141.2006.06317.x 16984390

[24] Royal College of Obstetricians and Gynecologists. Reducing the risk of venous thromboembolism during pregnancy and the puerperium: Green-top Guideline No 37A. 2015.

[25] Croles FN Nasserinejad K Duvekot JJ Kruip MJ Meijer K Leebeek FW. Pregnancy, thrombophilia, and the risk of a first venous thrombosis: systematic review and bayesian meta-analysis. BMJ. (2017) 359:j4452. 10.1136/bmj.j4452 29074563 PMC5657463

[26] Kevane B Donnelly J D'Alton M Cooley S Preston RJS Ní Ainle F. Risk factors for pregnancy-associated venous thromboembolism: a review. J Perinat Med. (2014) 42:417–25. 10.1515/jpm-2013-0207 24334422

[27] Jacobsen AF Skjeldestad FE Sandset PM. Ante- and postnatal risk factors of venous thrombosis: a hospital-based case-control study. J Thromb Haemost. (2008) 6:905–12. 10.1111/j.1538-7836.2008.02961.x 18363820

[28] O'Shaughnessy F O'Reilly D Ní Áinle F. Current opinion and emerging trends on the treatment, diagnosis, and prevention of pregnancy-associated venous thromboembolic disease: a review. Transl Res. (2020) 225:20–32. 10.1016/j.trsl.2020.06.004 32554071

[29] O'Shaughnessy F Donnelly JC Bennett K Damkier P Áinle FN Cleary BJ. Prevalence of postpartum venous thromboembolism risk factors in an Irish urban obstetric population. J Thromb Haemost. (2019) 17:1875–85. 10.1111/jth.14568 31309719

[30] Lindqvist PG Hellgren M. Obstetric thromboprophylaxis: the Swedish guidelines. Adv Hematol. (2011) 2011:157483. 10.1155/2011/157483 22162688 PMC3226316

[31] Dargaud Y Rugeri L Ninet J Negrier C Trzeciak MC. Management of pregnant women with increased risk of venous thrombosis. Int J Gynaecol Obstet. (2005) 90:203–7. 10.1016/j.ijgo.2005.05.003 15964002

[32] Dargaud Y Rugeri L Vergnes MC Arnuti B Miranda P Negrier C. A risk score for the management of pregnant women with increased risk of venous thromboembolism: a multicentre prospective study. Br J Haematol. (2009) 145:825–35. 10.1111/j.1365-2141.2009.07698.x 19388925

[33] Dargaud Y Rugeri L Fleury C Battie C Gaucherand P Huissoud C. Personalized thromboprophylaxis using a risk score for the management of pregnancies with high risk of thrombosis: a prospective clinical study. J Thromb Haemost. (2017) 15:897–906. 10.1111/jth.13660 28231636

[34] Chauleur C Gris JC Laporte S Rancon F Varlet M-N Decousus H. Use of the Delphi method to facilitate antithrombotics prescription during pregnancy. Thromb Res. (2010) 126:88–92. 10.1016/j.thromres.2010.01.012 20153880

[35] Chauleur C Quenet S Varlet M-N Seffert P Laporte S Decousus H. Feasibility of an easy-to-use risk score in the prevention of venous thromboembolism and placental vascular complications in pregnant women: a prospective cohort of 2,736 women. Thromb Res. (2008) 122:478–84. 10.1016/j.thromres.2007.12.020 18280547

[36] Chauleur C Gris J-C Laporte S Chapelle C Bertoletti L Equy V. Benefit of risk score-guided prophylaxis in pregnant women at risk of thrombotic events: a controlled before-and-after implementation study. Thromb Haemost. (2018) 118:1564–71. 10.1055/s-0038-1668524 30103244

[37] Schoenbeck D Nicolle A Newbegin K Hanley J Loughney AD. The use of a scoring system to guide thromboprophylaxis in a high-risk pregnant population. Thrombosis. (2011) 2011:652796. 10.1155/2011/652796 22084667 PMC3200277

[38] Testa S Passamonti SM Paoletti O Bucciarelli P Ronca E Riccardi A. The pregnancy health-care program for the prevention of venous thromboembolism in pregnancy. Intern Emerg Med. (2015) 10:129–34. 10.1007/s11739-014-1111-6 25078669

[39] Chau C Campagna J Vial M Rambeaud C Loundou A Bretelle F. Use of a personalized iterative score to evaluate risk of venous thromboembolism during pregnancy and puerperium. Int J Gynaecol Obstet. (2019) 144:277–82. 10.1002/ijgo.12754 30578681

[40] Lindqvist PG Kublikas M Dahlbäck B. Individual risk assessment of thrombosis in pregnancy. Acta Obstet Gynecol Scand. (2002) 81:412–6. 10.1034/j.1600-0412.2002.810507.x 12027814

[41] Lindqvist PG Torsson J Almqvist A Björgell O. Postpartum thromboembolism: severe events might be preventable using a new risk score model. Vasc Health Risk Manag. (2008) 4:1081–7. 10.2147/VHRM.S2831 19183756 PMC2605344

[42] Knight M Nair M Brocklehurst P Kenyon S Neilson J Shakespeare J. Examining the impact of introducing ICD-MM on observed trends in maternal mortality rates in the UK 2003–13. BMC Pregnancy Childbirth. (2016) 16:178. 10.1186/s12884-016-0959-z 27440079 PMC4955124

[43] Cantwell R Clutton-Brock T Cooper G Dawson A Drife J Garrod D. Saving mothers' lives: reviewing maternal deaths to make motherhood safer: 2006–2008. the eighth report of the confidential enquiries into maternal deaths in the United Kingdom. BJOG. (2011) 118(Suppl 1):1–203. 10.1111/j.1471-0528.2010.02847.x 21356004

[44] Sultan AA West J Grainge MJ Riley RD Tata LJ Stephansson O. Development and validation of risk prediction model for venous thromboembolism in postpartum women: multinational cohort study. BMJ. (2016) 355:i6253. 10.1136/bmj.i6253 27919934 PMC5137302

[45] Fuchs F Benhamou D. [Post-partum management after cesarean delivery. Guidelines for clinical practice]. J Gynecol Obstet Biol Reprod. (2015) 44:1111–7. 10.1016/j.jgyn.2015.09.020 26527019

[46] American College of Obstetricians and Gynecologists' Committee on Practice Bulletins—Obstetrics. ACOG practice bulletin no. 196: thromboembolism in pregnancy. Obstet Gynecol. (2018) 132:e1–17. 10.1097/AOG.0000000000002706 29939938

[47] Bates SM Rajasekhar A Middeldorp S McLintock C Rodger MA James AH. American society of hematology 2018 guidelines for management of venous thromboembolism: venous thromboembolism in the context of pregnancy. Blood Adv. (2018) 2:3317–59. 10.1182/bloodadvances.2018024802 30482767 PMC6258928

[48] Crowley MP Noone C Higgins JR O'Shea S. A Multicentre Study of Thromboprophylaxis in Pregnancy. Ir Med J. (2017) 110:567. 28737308

[49] Friedman AM Ananth CV Lu Y-S D'Alton ME Wright JD. Underuse of postcesarean thromboembolism prophylaxis. Obstet Gynecol. (2013) 122:1197–204. 10.1097/AOG.0000000000000007 24201686

[50] D'Alton ME Friedman AM Smiley RM Montgomery DM Paidas MJ D'Oria R. National partnership for maternal safety: consensus bundle on venous thromboembolism. Obstet Gynecol. (2016) 128:688–98. 10.1097/AOG.0000000000001579 27607857

[51] Borab ZM Lanni MA Tecce MG Pannucci CJ Fischer JP. Use of computerized clinical decision support systems to prevent venous thromboembolism in surgical patients: a systematic review and meta-analysis. JAMA Surg. (2017) 152:638-45. 10.1001/jamasurg.2017.0131 28297002 PMC5831455

